# Intrathecal Morphine Versus Other Techniques for Postoperative Pain Management in the Context of Multimodal Analgesia: A Meta-Analysis

**DOI:** 10.3390/ph18040512

**Published:** 2025-03-31

**Authors:** Arron W. Gibson, Niamh E. Cooper, Eric Albrecht, Patrice Forget

**Affiliations:** 1 School of Medicine and Medical Sciences, University of Aberdeen, Aberdeen AB25 2ZD, UK; niamhecooper@icloud.com; 2 Department of Anesthesia, University Hospital of Lausanne, 1011 Lausanne, Switzerland; 3 Pain and Opioids After Surgery (PANDOS) European Society of Anaesthesiology and Intensive Care (ESAIC) Research Group, 1000 Brussels, Belgium; 4 Aberdeen Centre for Arthritis and Musculoskeletal Health (Epidemiology Group), Institute of Applied Health Sciences and Nutrition, School of Medicine and Medical Sciences, University of Aberdeen, Aberdeen AB25 2ZD, UK; 5 Department of Anaesthesia, NHS Grampian, Aberdeen AB25 2ZD, UK; 6 IMAGINE UR UM 103, Anaesthesia Critical Care, Emergency and Pain Medicine Division, Nîmes University Hospital, Montpellier University, 30900 Nîmes, France

**Keywords:** intrathecal morphine, multimodal analgesia, postoperative pain

## Abstract

**Objective**: Intrathecal morphine (ITM) has been administered in recent years to provide postoperative pain control in non-obstetric surgery; however, current research has limited consideration of the recommendations for regular, basic analgesia from clinical guidelines when exploring its efficacy. This systematic review and meta-analysis aimed to compare ITM against alternative methods of analgesia in the presence of multimodal analgesia, for reducing pain scores within the first 24 h postoperatively. Secondary outcomes included postoperative opioid consumption, incidence of opioid-related effects, and time to mobilisation. **Methods**: Database searches and screening identified 11 trials for inclusion in this review. Pain scores were compared by meta-analysis at 6, 12, and 24 h postoperatively at rest and on movement, with sub-analysis of systemic versus regional techniques. **Results**: The data found no significant difference between ITM and active comparators for reducing pain scores at rest or on movement at any of the time intervals explored. Sub-analysis demonstrated that regional techniques may provide superior analgesia at 24 h at rest (MD = −1.19; 95% CI [−1.73, −0.66], *p* < 0.001, I^2^ = 0%) and on movement (MD = 1.27 [0.44, 2.10], *p* = 0.003, I^2^ = 0%). Cumulative opioid consumption was reduced in ITM groups (MD = −11.61 [−18.73, −4.50], *p* = 0.001, I^2^ = 95%), with significantly increased risk of pruritus (*p* < 0.001) but not nausea and vomiting (*p* = 0.93). There was no evidence of respiratory depression. **Conclusions**: This meta-analysis was unable to demonstrate any significant benefit to postoperative pain relief with the use of ITM but may suggest that it is as a viable option compared to other active modalities. However, this meta-analysis was limited by a low quantity and quality of data from which to draw conclusions and demonstrated high statistical fragility. We believe this highlights a significant gap in the current literature on ITM.

## 1. Introduction

Intrathecal morphine (ITM), or spinal morphine, is a method of analgesia used to reduce postoperative pain via the delivery of morphine directly into the cerebrospinal fluid (CSF). Pain after surgery is highly prevalent, with the Perioperative Quality Improvement Programme (PQIP) Annual Report 2021 [1] identifying 1 in 5 patients suffering from severe pain in the first 24 h postoperatively. This may worsen both subjective and objective outcomes for patients; therefore, improving pain should be a priority for clinicians going forward. ITM may provide a potent means by which to control postoperative pain in the future.

Despite the potential benefits, certain downsides need to be contemplated when considering ITM in practice. It may be difficult to gauge the subjective impact on patient experiences from receiving such an invasive procedure. Intrathecal opioids may lead to direct harm as they are associated with significant side effects such as respiratory depression, nausea and vomiting, pruritus, and tolerance. A ‘ceiling analgesic effect’ of ITM has been described whereby the risk of opioid-related side effects is perceived to outweigh the pain-relieving benefits—although the dose at which this occurs varies depending on the type of surgery being performed [2]. In general, they found doses >300 micrograms (μg) seemed to cause an increased incidence of side effects. However, a 2022 review [3] found a dose of 100 μg is the threshold for increased postoperative nausea and vomiting in relation to lower limb arthroplasty surgery, whereby increasing the dose beyond this point correlated with a linear rise in side effects. A notable further phenomenon, opioid-induced hyperalgesia, may mean that for some, opioids may have a short-acting analgesic effect, but a long-acting and counterproductive, hyperalgesic effect [4].

The use of ITM is well established in obstetric surgery, with Prospect Working Group endorsed guidelines in 2020 recommending intrathecal morphine as a component of caesarean section analgesia, in combination with spinal anaesthesia [5]. They also discuss the recommendation for intraoperative and postoperative paracetamol and NSAID, reserving opioids for breakthrough pain.

Research from groups in recent years has provided great evidence to demonstrate that ITM represents a potent and viable option for postoperative analgesia in non-obstetric surgery [3]. However, PROSPECT themselves have identified a limitation to pain research whereby many randomised controlled trials (RCTs) assess the efficacy of an intervention compared to a placebo group and utilise rescue opioids for analgesia. In general, the literature thus far regarding ITM has relied heavily on conclusions made from placebo and negative controls, reducing its relevance and transferability to the clinician. PROSPECT also recommend the use of a regular, basic analgesia regimen in the postoperative period, comprising paracetamol plus another non-opioid (non-steroidal anti-inflammatory drugs (NSAID) or cycloxygenase-2 (COX-2) specific inhibitor). The use of opioid-sparing techniques and multimodal analgesia is further reinforced by the Faculty of Pain Medicine publication [6], “Surgery and Opioids: Best Practice Guidelines”. This is in line with the current National Institute for Health and Care Excellence (NICE) Analgesia Guidelines for Mild to Moderate Pain, widely accepted in modern practice, which recommends a stepwise approach starting with paracetamol before progressing to other non-opioids or combinations before using opioid therapy [7]. Again, this is something that appears to vary greatly in the available literature on this topic.

We aimed to compare ITM against alternative analgesic options, in the presence of multimodal analgesia, thus addressing an apparent a gap in the literature and improving clinical relevance for modern practice.

## 2. Methods

This systematic review was carried out in accordance with the PRISMA 2020 Statement Checklist [8] and registered with PROSPERO International Prospective Register of Systematic Reviews on 21 February 2023 (registration number: CRD42023399093).

**Figure 1:**
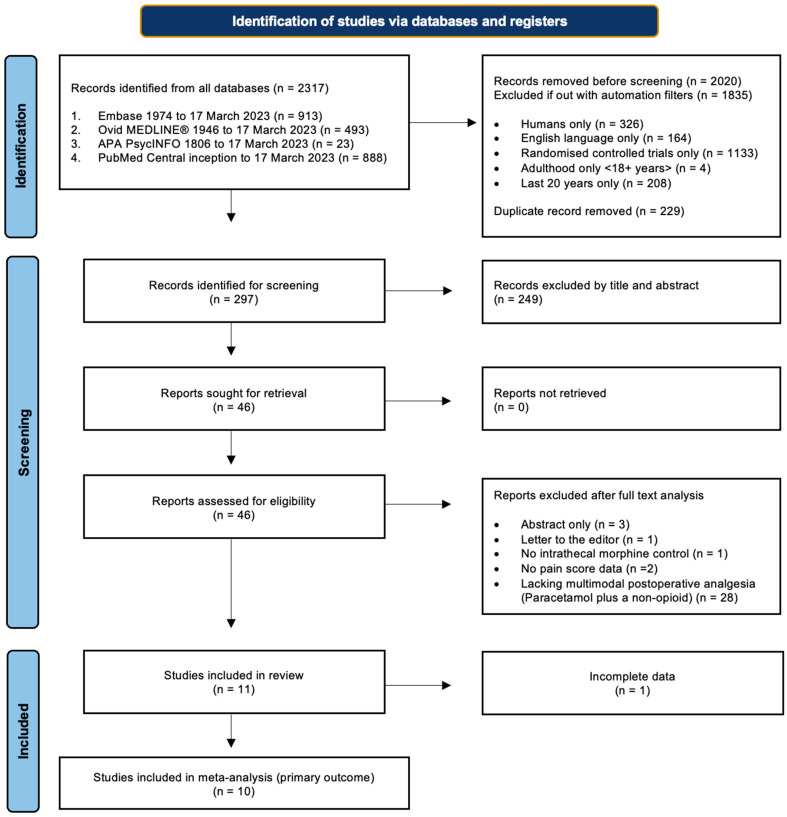
Study selection. Flow diagram demonstrating the study selection process, adapted from PRISMA guidelines [8].

Initial searches were conducted on electronic databases Ovid MEDLINE, Embase, APA PsychInfo, and PubMed from the date of inception to the date of the search on 17 March 2023. A comprehensive list of search terms and limiters can be found in Appendix A.1. The primary search results were then exported and screened by two researchers independently, and references were checked at each stage for discrepancies. Disagreements were resolved by discussion, but if no consensus was reached, they were adjudicated by a third, impartial researcher. Primary search results were exported to reference managing software (RefWorks Proquest and Endnote 20.6) before duplicates were removed. References were first screened by title and abstract, and then again by detailed analysis of the full texts (Figure 1).

RCTs conducted from 2003 to 2023, which contained only adult (>18 years of age) human participants undergoing surgery of any kind, were included. To be considered, studies must use numerical pain scores to compare the efficacy of ITM in reducing postoperative pain. The active comparator must be administered within the operating theatre immediately before, during or after the surgical procedure. Studies had to include a clear record of regular use of at least two regular non-opioid analgesics in which one was paracetamol (or equivalent), and the other an NSAID or COX-2 inhibitor, as per the PROSPECT standards of multimodal analgesia [9]. The use of spinal anaesthesia was not controlled.

Studies were excluded if they included paediatric patients, investigated the use of epidural morphine, did not clearly describe regular use of multimodal analgesia, or did not report administration of regular PRN analgesia. The criteria for inclusion can be found in the PICOS in Appendix A.1.

Pre-defined outcomes were extracted from the chosen papers independently in accordance with internationally accepted standards of the PROSPECT methodology [9] by two researchers and discrepancies were cross-referenced to check for inaccuracies before the analysis of the data. For the primary outcome of pain management, pain scores at rest and on movement were extracted at 6, 12 and 24 h postoperatively as mean values with standard deviation (SD). Postoperative opioid consumption was extracted for the first 24 h postoperatively as mean and SD. Opioid doses were converted to IV morphine equivalent doses; specifically, 1mg of IV piritramide was considered equivalent to 0.7 mg of IV morphine. Opioid-related side effects, including postoperative pruritis, nausea and vomiting, and any respiratory depression, were extracted as incidence over the first 24 h after the operation. Time to mobilisation was extracted when the number of hours to first mobilisation postoperatively was specified.

Where possible, data were collected directly from tables and text in the form of mean with SD. On occasions where data were presented graphically, and data extraction was completed through Plot Digitiser [10]—a free, online software (https://plotdigitizer.sourceforge.net) used for extracting raw data from graphs and verified for scientific use [11]. Where data were unavailable, corresponding authors were contacted twice to request missing data points. If this information was not given, median and interquartile range (IQR) were converted to mean and SD, respectively, using the sample size-dependent conversion tool [12], as recommended in the Cochrane Handbook for Systematic Reviews of Interventions, Chapter 6.5.2.5 [13].

Data points from the VAS pain scale, which utilised a scale from 0 to 100mm, were directly converted to an eleven-point, 0 (no pain) to 10 (worst imaginable pain) scale. The PROSPECT methodology [9] suggests a mean difference of 10mm on a VAS scale should be considered a statistically significant effect. As such, an effect size of >1.0 in the meta-analysis was considered a statistically significant change.

For this review, NRS during mobilisation on day 1 and day 2 was assumed to be 12 and 24 h, respectively, postoperatively [14]. Likewise, the first postoperative day, morning and evening, were assumed to be 12 and 24 h, respectively [15]. Sensitivityanalyses were performed.

The evidence used for each outcome was assessed for quality, according to GRADE (Grading of Recommendations, Assessment, Development and Evaluations), following guidance from Chapter 14 in the Cochrane Handbook for Systematic Reviews of Interventions version 6.3 [16]. GRADEpro software [17] was used for the assessment. Explanations for the judgement were included as part of the summary of findings table (Table 1). Heterogeneity (I^2^) was interpreted according to the Cochrane Handbook, Chapter 10.10.2 [18], whereby low heterogeneity is considered ≤40%, moderate heterogeneity 30–60%, substantial heterogeneity 50–90%, and considerable heterogeneity ≥75%. Their respective *p*-values were considered when making this judgement, alongside the number of studies represented and the likely magnitude of the effect. The risk of bias in the included studies was also contemplated in the GRADE assessment.

The methodology for each trial was assessed for quality by two researchers using the Cochrane Revised Risk of Bias (RoB2) Excel Tool for randomised controlled trials [19]. Any discrepancies were adjudicated by a third, impartial researcher. Each domain was assessed as high risk, low risk, or raises concerns and an overall assessment was then attached to the study in question (Figure 1). Funnel plots were generated to graphically assess for risk of bias [20].

Meta-analyses of all listed outcomes were conducted on Review Manager Version 5.4 [21]. Relative heterogeneity (≥40%) [18] was present in most outcomes. The random effects model was used for the calculation of the mean difference in continuous variables with effect sizes, 95% confidence interval and *p*-values calculated to signify statistical significance.

Sub-analysis investigated the extent to which different active comparators influenced the primary outcome, and to begin addressing heterogeneity observed in the study data. Study data were grouped based on the active comparator compared to ITM for each of the time points considered. Any active comparator affecting the whole body via the circulation was considered as “systemic analgesia”, while all those affecting an isolated region or nerve were considered “regional anaesthesia”, and so included local infiltrate analgesia techniques.

Sensitivity analyses were subsequently performed by systematically excluding each data set to assess statistical fragility. Sensitivities analyses also explored if controlling, or not controlling, the use of spinal anaesthesia in ITM and active comparator groups significantly altered the observed effects by excluding studies that featured the use of spinal anaesthesia in only ITM groups. Finally, sensitivity analyses explored if there was an impact on the effect of the analysis if only studies published in the last 10 years were considered, in comparison to those from the last 20 years.

## 3. Results

The literature search identified 2317 records, of which 297 were brought forward for screening. From this, 11 RCTs, totalling 637 participants, were eligible for inclusion in this review, with one excluded from the primary outcome due to a lack of data spread [22] (Figure 1). The characteristics of each study are viewable in Table 2.

Three studies compared intrathecal morphine to systemic analgesia using an intravenous opioid bolus [26,28,29]. The remaining eight studies utilised regional techniques, including the following: femoral nerve block [22,24,30], lumbar plexus block [14], psoas compartment block [26], local infiltration analgesia [15,27], and periarticular multimodal injection [25]. Nine studies employed the use of spinal anaesthesia, the control of which is depicted in study characteristics Table 2.

### 3.1. Primary Outcome

Seven studies [15,23,25,27,28,29,30] involving 350 patients reported pain scores at rest at 6, 12 or 24 h. Data were inputted for meta-analysis (Appendix A.2), calculating mean difference (MD) with a 95% confidence interval (95% CI). At each time point, there was no statically significant different inpatient pain scores between ITM and active comparator groups: 6 h (−0.42 MD [95% CI −2.12, 1.28], *p* = 0.63; I^2^ = 88%, *p* <0.001), 12 h (−0.35 [−1.38, 0.68], *p* = 0.54; I^2^ = 75%, *p* = 0.003), 24 h (0.07 [−0.93, 1.07], *p* = 0.89; I^2^ = 84%, *p* < 0.001).

Seven studies [14,15,24,27,28,29,30] involving 423 patients reported pain scores on movement at 6, 12 or 24 h. Pain scores on movement (Appendix A.3) also showed no significant difference at each time interval between ITM and active comparator patient groups: 6 h (−0.04 [−1.10, 1.02], *p* = 0.94, I^2^ = 71%, *p* = 0.03), 12 h (−0.64 [−1.56, 0.28], *p* = 0.17, I^2^ = 74%, *p* = 0.004), and 24 h (0.61 [−0.89, 2.12], *p* = 0.43, I^2^ = 94%, *p* < 0.001). Significant heterogeneity was seen at each interval.

Two studies using systemic comparators had data for pain on rest [26,28,29] (Appendix A.4). One study [28] reported data at 12 h (−0.60 [−1.97, 0.77], *p* = 0.39, I^2^ = n/a) and two studies [26,28] reported data at 24 h (−1.19 [−1.73, −0.66], *p* < 0.001, I^2^ = 0%, *p* = 0.36). Regional comparators to ITM were tested at rest by five studies [15,23,25,27,30] (Appendix A.5). Data at 6 h reported (−0.42 [−2.12, 1.28], *p* = 0.63, I^2^ = 88%, *p* < 0.001) [15,23,30], 12 h reported (−0.29 [−1.56, 0.98], *p* = 0.65, I^2^ = 81%, *p* = 0.001) [15,23,25,30], and 24 h reported (0.62 [−0.50, 1.74], *p* = 0.28, I^2^ = 81%, *p* = 0.006) [15,23,25,27,30]. Sensitivity analyses (Sup to eliminate studies older than 10 years found (1.44 [0.38, 2.50], *p* = 0.008, I^2^ = 1%, *p* = 0.31) at 24 h, favouring regional analgesia (based on two RCTs). Sensitivity analysis was not applicable for systemic comparators. Detailed results can be found as Supplementary Information.

Two studies [28,29] using systemic comparators had data for pain on movement (Appendix A.6). No studies reported pain data at 6 h, data at 12 h reported (−0.20 [−1.83, 1.43], *p* = 0.81, I^2^ = n/a) [28], and data for 24 h showed (0.82 [−0.89, 2.53], *p* = 0.35, I^2^ = 63%, *p* = 0.1) [28,29]. A sensitivity analysis for the last 10 years was not required. Five studies using regional comparators had data for pain on movement (Appendix A.7). Data at 6 h showed (−0.04 [−1.10, 1.02], *p* = 0.94, I^2^ = 71%, *p* = 0.03 [15,24,30], data at 12 h reported (−0.72 [−1.76, 0.33], *p* = 0.18, I^2^ = 78%, *p* = 0.003) [15,24,30], and data for 24 h found (0.59 [−1.22, 2.39], *p* = 0.52, I^2^ = 93%, *p* < 0.001) [14,15,24,27,30]. Full results are available in Supplementary Information. Sensitivity analysis eliminating opposing pain on movement data [24] produced homogeneous results (I^2^ = 0%, *p* = 0.76) with overall MD 1.28 [0.58, 1.98], *p* <0.001, therefore in favour of regional techniques at 24 h. Additional sensitivity analyses for RCTs published from 2013 onwards also produced results favouring regional techniques: 1.27 [0.44, 2.10], *p* = 0.003, I^2^ = 0%, *p* = 0.56.

### 3.2. Secondary Outcome—Cumulative Opioid Consumption

Four studies [23,24,26,29] involving 298 patients reported cumulative opioids within the first 24 h (Appendix A.8). All data were converted to milligrams (mg) of IV morphine. Analysis showed patients who received ITM subsequently consumed less opioid (mg) in the first 24 h postoperatively: −11.61 [−18.73, −4.50], *p* = 0.001, I^2^ = 95%; *p* < 0.001.

### 3.3. Secondary Outcome—Opioid-Related Side Effects (Appendix A.9)

Three studies [14,15,29] involving 248 patients contained data regarding the incidence of nausea and vomiting over the first 24 h. The meta-analysis did not show a statistically significant increase in the relative risk of nausea and vomiting in patients receiving ITM in comparison to alternative modalities: 1.02 [0.68, 1.53], *p* = 0.93 (I^2^ = 7%, *p* = 0.34).

Two studies [15,29] involving 190 patients recorded incidence of pruritus over 24 h with meta-analysis suggesting lower incidence in patients receiving the comparator compared to ITM: 3.55 [1.92, 6.56], *p* < 0.001 (I^2^ = 0%, *p* = 0.36).

Six studies [23,24,25,26,27,29] involving 394 patients measured the incidence of respiratory depression, all of which found no cases of respiratory depression in either study group.

Time to mobilisation, in hours, was only recorded by one study [26]. Therefore, it was not possible to conduct a meta-analysis for this secondary outcome.

### 3.4. Risk of Bias and Quality Assessment

**Figure 2:**
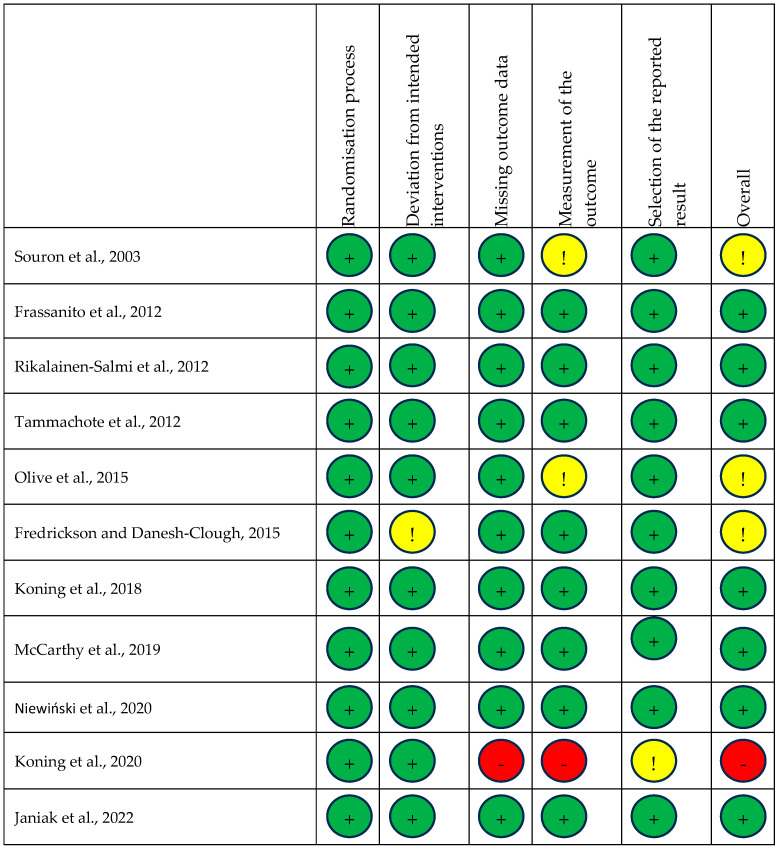
Risk of bias summary. Risk of bias summary using Cochrane RoB 2 tool. Green, yellow, and red circles depict low, unclear, and high bias for each domain, respectively, according to researcher judgement [14,15,22,23,24,25,26,27,28,29,30].

Each trial was also assessed for risk of bias, with a summary shown in Figure 2. One study was assessed as high risk of bias [29], three as having some concerns [14,22,23], and seven as low risk [15,23,24,25,26,27,28,30].

The quality of evidence (GRADE [17]) was assessed and is depicted in Table 1, ‘Summary of Findings’, including the rationale for each assessment. The quality of evidence ranged from low to very low due to high heterogeneity in study data, small sample size, large confidence interval range, and containing data from studies judged as high risk of bias.

Funnel plots for each outcome on RevMan [21] were viewed and assessed to judge the risk of publication bias. Visual analysis of plots found no obvious asymmetry for each outcome, implying the likelihood of imprecise findings was low for the studies included in this review. However, this is with limited accuracy given the high level of heterogeneity and low number of data sets in each analysis.

## 4. Discussion

This meta-analysis found no statistical difference in reducing postoperative pain between ITM and active comparators within the first 24 h at rest or on movement. The quality of evidence for these ranged from very low to moderate, and the heterogeneity was generally high, adding significant difficulty when drawing conclusions based on these data. Subsequent sensitivity analyses indicated relative statistical fragility and significant reliance on some of the included studies for the overall result. This suggests there is insufficient data to support the use of ITM ahead of other equivalent techniques; however, it is worth considering that ITM may be as effective as these active comparators and could represent a valid option for the clinician’s toolkit.

A sub-group analysis was performed to ascertain whether there was a difference when comparing ITM against systemic comparators versus regional and local comparators. This aimed to explore the significant heterogeneity in the meta-analyses of the primary outcomes. A comparison of ITM to systemic analgesia found ITM improved postoperative pain at rest at 24 h, but not on movement. A comparison of ITM and regional techniques did not find a significant reduction in pain scores at 24 h postoperatively, at rest or on movement.

Sensitivity analyses of trials depicting pain at rest from the last 10 years (2013 onwards) found pain in the comparator group was lower at 6 h; however, only based on one trial. Findings at 12 h were statistically unchanged. Notably, at 24 h, the pain scores were lower at rest in the group receiving regional analgesia compared to ITM. Sensitivity analyses considering pain on movement also showed reduced pain at 6 h in the comparator group; however, based on only one eligible study. Pain on movement at 24 h appeared to improve in patients receiving the comparator, with lower pain scores in the regional groups compared to ITM. A 10-year sensitivity analysis comparing systemic analgesia and ITM at rest and on movement was not required due to study data being within this period.

Patients receiving ITM used fewer opioids in the first 24 h postoperatively compared to those in an active comparator group. This was supported by sensitivity analyses for studies within the last 10 years. While it may be tempting to consider this as evidence of an opioid-sparing effect, it might be prudent to consider that ITM is much more potent than IV and oral morphine. There is some speculation over exactly how much more potent ITM is compared to other routes, but this could potentially account for some, or even all, of the difference in overall opioid exposure seen for the ITM intervention groups.

There were no instances of respiratory depression reported in any studies included in this review. This means we were unable to calculate a relative risk ratio from our results and can conclude it is likely a relatively low risk. This is in keeping with recent research [3] that found no increased relative risk of respiratory depression and suggested patients receiving an intrathecal dose of 100 μg may only need standard postoperative care due to the low risk. They also proposed that fear of respiratory depression was based on older research when larger doses were more common. Further evidence found a 1% incidence of respiratory depression in doses <0.3 mg, which rose to 9% in doses at or greater than 0.3 mg, demonstrating risk closely associated with the dose [31]. Supporting this is research exploring a case series of patients receiving 20-fold increased doses due to prescribing errors and found respiratory depression to be a common and serious side effect [32].

Our meta-analysis indicated that pruritis was the most frequent opioid-related side effect associated with ITM and was graded moderate quality of evidence; however, it should be noted that this is based on a large effect from a single study which also used large doses of morphine, which may have influenced the overall incidence. The analysis found no increase in the relative risk of postoperative nausea and vomiting; however, this was based on very low quality of evidence. We found very little data on the effect of time on mobilisation. In general, there has been limited exploration of this in reviews on ITM.

It is important to note that this review had significant limitations that reduced the quality of evidence. Firstly, there was limited data due to very few RCTs fulfilling the defined PICOS criteria. There were also instances where data were unusable due to a lack of data spread [22], or results without clear identification of time (specifically regarding the incidence of opioid-related side effects), further compressing the data set for the meta-analysis. Secondly, there was high heterogeneity between the studies included in this meta-analysis due to differences in methodology, active comparators, and dose of ITM. We considered multiple types of surgery together without consideration for the difference in analgesic needs for each of these surgeries. It would have been a useful comparison to consider this solely within the context of orthopaedic procedures as a separate entity; however, there were insufficient studies available fulfilling our criteria to facilitate this sub-analysis as part of this review. Thirdly, one study was found to be at high risk of bias [29] and another to have some concerns around bias [23]—it may be difficult to know if this impacted the result of the meta-analysis. Fourthly, a key concern associated with opioids is the risk of urinary retention, which we did not investigate within the scope of this study. Lastly, there may have been significant limitations in the measurement of respiratory depression, as there were no set criteria for what was to be defined as respiratory depression (e.g., based on hypercapnia or respiratory rate) in the RCTs included in our review. It has also been suggested that the term “respiratory depression” inadequately describes the full picture and that “opioid-induced ventilatory impairment” may better describe the mechanisms at play [33]. In addition, the studies were often not specifically designed to detect respiratory depression, and the likelihood of missed events is hard to gauge. Another limitation is that we have not included any studies from 2024 onwards.

In conclusion, there is insufficient evidence to recommend ITM instead of an alternative analgesic option. This also means that ITM could be at least as effective as other options and could represent a viable option for clinicians. Due to the overall low quantity and quality of evidence, it is unlikely to be possible to conclude, with a high level of certainty, if ITM provides better analgesia in the first 24 h postoperatively, compared to alternative methods of analgesia when given alongside regular, basic multimodal analgesia. We appreciate ITM reduces opioid consumption postoperatively, but not necessarily overall exposure to opioids, while the risk of pruritus may be increased in patients receiving ITM compared to an alternative method. There was no evidence of differences between groups regarding nausea and vomiting and respiratory depression risk. We believe this analysis demonstrates there is inadequate evidence to support the use of ITM ahead of alternative techniques in a clinical setting where regional analgesia can be used, thus representing a significant gap in the literature. This is especially true given the invasiveness and potential harm associated with intrathecal morphine administration. Ultimately, to reach an informed decision on using ITM in real-world practice, more research is required. These should ideally be surgery-specific and use standardised methodologies (like PROSPECT) to compare specific alternative modalities.

## Figures and Tables

**Table 1:** Summary of findings.

*Primary Outcome*	Mean Difference [95% CI]	*p*-Value	I^2^ (*p*-Value)	*n* [Studies]	GRADE ^EXPLANATION^
*Postoperative pain at rest at 6 h*	−0.42 [−2.12, 1.28]	0.63	88% (<0.001)	158 [3]	Very low ⊕⊖⊖⊖ ^a,b,c^
*Postoperative pain at rest at 12 h*	−0.35 [−1.38, 0.68]	0.51	75% (0.003)	251 [5]	Low ⊕⊕⊖⊖ ^a^
*Postoperative pain at rest at 24 h*	0.07 [−0.93, 1.07]	0.89	84% (<0.001)	350 [7]	Low ⊕⊕⊖⊖ ^a^
*Postoperative pain on movement at 6 h*	−0.04 [−1.10, 1.02]	0.94	71% (0.03)	157 [3]	Very low ⊕⊖⊖⊖ ^b,c,d^
*Postoperative pain on movement at 12 h*	−0.64 [−1.56, 0.28]	0.17	74% (0.004)	243 [5]	Low ⊕⊕⊖⊖ ^b,d^
*Postoperative pain on movement at 24 h*	0.61 [−0.89, 2.12]	0.43	94% (<0.001)	423 [7]	Very low ⊕⊖⊖⊖ ^a,b,e^
***Secondary Outcomes***	**Mean Difference [95% CI]**	***p*-Value**	**I^2^ (*p*-Value)**	***n*** **[Studies]**	**GRADE ^EXPLANATION^**
*Opioid consumption (mg) 24 h postoperatively*	−11.61 [−18.73, −4.50]	0.001	95% (<0.001)	298 [4]	Very low ⊕⊖⊖⊖ ^a,e^
*Time to mobilisation (hours)*	Insufficient number of eligible studies.				
***Secondary Outcomes***	**Risk Ratio [95% CI]**	***p*-Value**	**I^2^ (*p*-Value)**	***n*** **[Studies]**	**GRADE ^EXPLANATION^**
*Nausea and vomiting: incidence 24 h postoperatively*	1.02 [0.68, 1.53]	0.93	7% (0.34)	248 [3]	Low ⊕⊕⊖⊖ ^e^
*Pruritus: incidence 24 h postoperatively*	3.55 [1.92, 6.56]	<0.001	0% (0.36)	190 [2]	Very low ⊕⊖⊖⊖ ^c,e^
*Respiratory depression: incidence 24 h postoperatively*	Not estimable.				

Table displaying overall effect estimates for each outcome comparing intrathecal morphine to an active comparator, adapted from the Cochrane Handbook for Systematic Reviews of Interventions [16]. Abbreviations: CI = confidence interval; *n* = number of participants; GRADE = Grading of Recommendations, Assessment, Development, and Evaluations; ITM = intrathecal morphine; mg = milligrammes. ^a^ Considerable heterogeneity (≥75%). ^b^ Extremities of upper and lower values of the 95% CI mean the true effect may favour either ITM or the comparator; therefore, the clinical decision may differ based on these values. ^c^ Low number of studies. ^d^ Substantial heterogeneity (≥50%). ^e^ Included data from a study considered high risk of bias.

**Table 2:** Characteristics of included studies.

Reference	Locations	Surgery	Mean Age (SD)	Gender Ratio (M/F)	ITM Dose	Active Comparator	Spinal Anaesthesia	Post-OP Multi-Modal Analgesia
Souron et al., 2003 [23]	France	Primary hip arthroplasty	ITM = 66.8 (13.1) C = 67.7 (12)	ITM = 12/14 C = 9/18	100 µg	Psoas-compartment block	n/a	IV paracetamol IV ketoprofen
Frassanito et al., 2012 [24]	Italy	Total knee arthroplasty	ITM = 67.4 (8.5) C = 68 (5.8)	ITM = 19/6 C = 14/12	100 µg	Single-shot ultrasound guided femoral nerve block	Bupivacaine 15 mg	IV paracetamol IV ketorolac
Rikalainen-Salmi et al., 2012 [15]	Finland	Total hip arthroplasty	ITM = 66 (6.3) LIA = 65 (8.7)	ITM = 11/17 C =10/19	100 µg	Local infiltration analgesia	Bupivacaine 16 mg	PO paracetamol PO ibuprofen
Tammachote et al., 2012 [25]	Thailand	Total knee arthroplasty	ITM = 69 (8) C = 70 (7)	ITM = 8/20 C =3/26	200 µg	Periarticular multimodal drug injection	0.5% bupivacaine 2.5 mL	Paracetamol Amitriptyline Naproxen
Olive et al., 2015 [22]	Australia	Total knee joint replacement	ITM = 70.6 C = 70.3	ITM = 10/17 C = 12/16	1750 µg	Continuous femoral nerve block	0.5% bupivacaine 3.5 mL	PO paracetamol PO celecoxib
Fredrickson and Danesh-Clough, 2015 [14]	New Zealand	Total hip joint replacement	ITM =62.6 (9.4) C = 63.2 (7.2)	ITM = 9/14 C =12/15	100 µg	Patient controlled continuous femoral nerve bock	0.5% bupivacaine 2 mL (ITM only)	PO paracetamol PO diclofenac (sustained release)
Koning et al., 2018 [26]	Netherlands	Laparoscopic segmental colonic resection	ITM = 66.1 (7.8) C = 67.9 (11.1)	ITM = 18/9 C = 15/14	300 µg (240 µg if >70 yo)	IV piritramide bolus	Bupivacaine 12.5 mg; or 10 mg if age >75 (ITM only)	Paracetamol Metamizole
McCarthy et al., 2019 [27]	Ireland	Total knee arthroplasty	ITM = 64.3 (8.9) C = 66.0 (9.8	ITM = 12/10 C = 12/9	300 µg	Local infiltration analgesia	0.5% bupivacaine 15 mg if <70 kg BW; 17.5 mg if >70 kg BW	PO paracetamol PO diclofenac sodium
Niewiński et al., 2020 [28]	Poland	Hepatectomy	ITM = 60 (10.5) C = 55 (12.1)	ITM = 9/9 C = 6/12	400 µg	IV morphine bolus	n/a	Paracetamol Dexketoprofen
Koning et al., 2020 [29]	Netherlands	Robot-assisted radical prostatectomy	ITM = 66 (5.6) C = 65.3 (7.8)	ITM = 66/0 C = 71/0	300 µg	IV morphine bolus	Bupivacaine 12.5 mg (ITM only)	IV paracetamol IV metamizole
Janiak et al., 2022 [30]	Poland	Total knee arthroplasty	ITM = 68 (11.9) C = 67.5 (9.7)	ITM = 23/3 C =23/3	100 µg	Single-shot femoral nerve block	0.5% bupivacaine 15 mg	IV paracetamol IV ketoprofen

**Figure A1:**
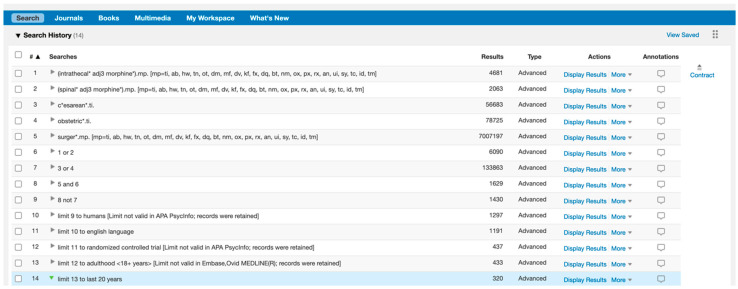
Search Strategy.

**Figure A2:**
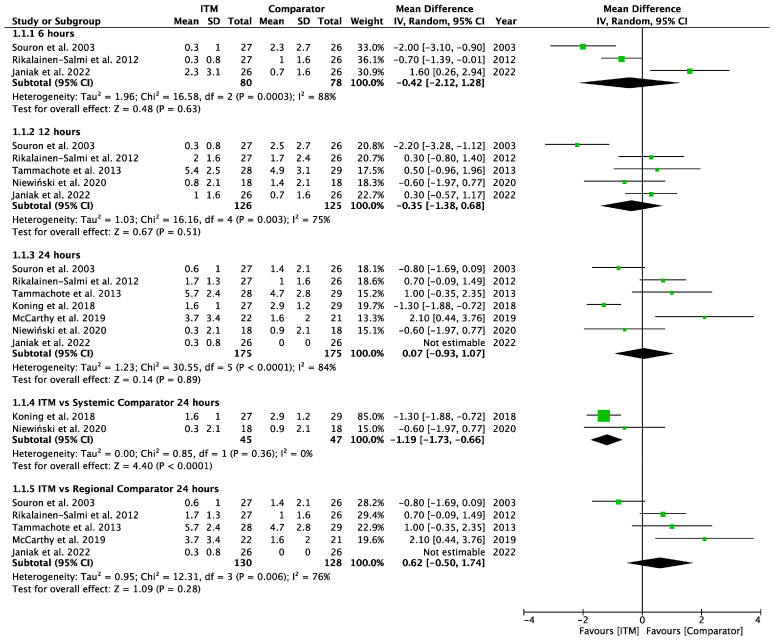
Postoperative Pain at Rest [15,23,25,26,28,29,30].

**Figure A3:**
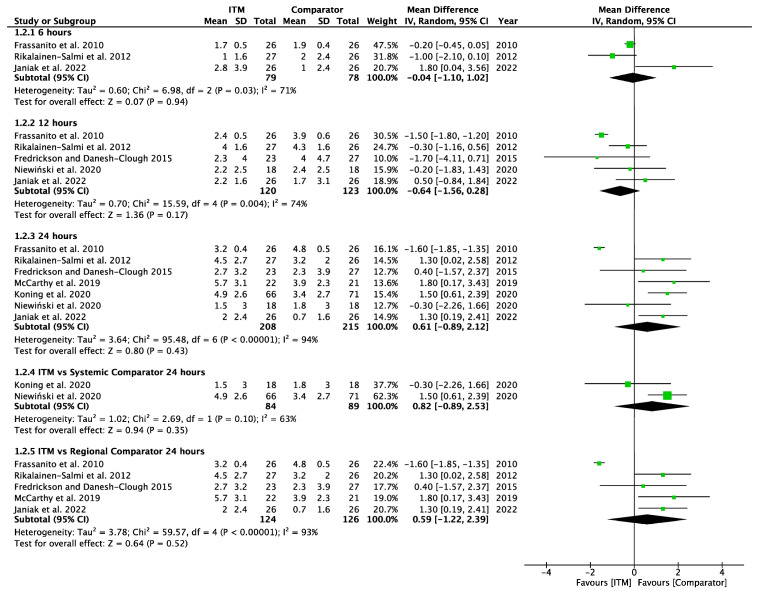
Postoperative Pain on Movement [14,15,24,25,26,27,29].

**Figure A4:**
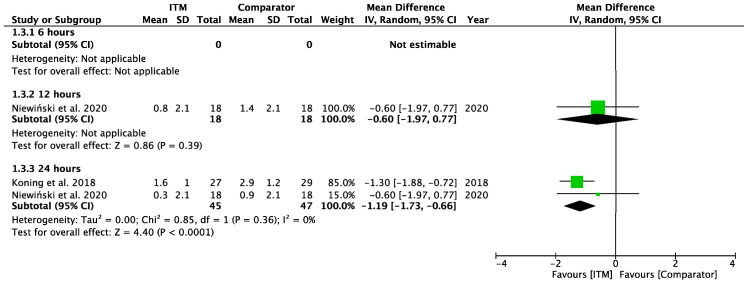
Postoperative Pain at Rest—Systemic Comparators [26,28].

**Figure A5:**
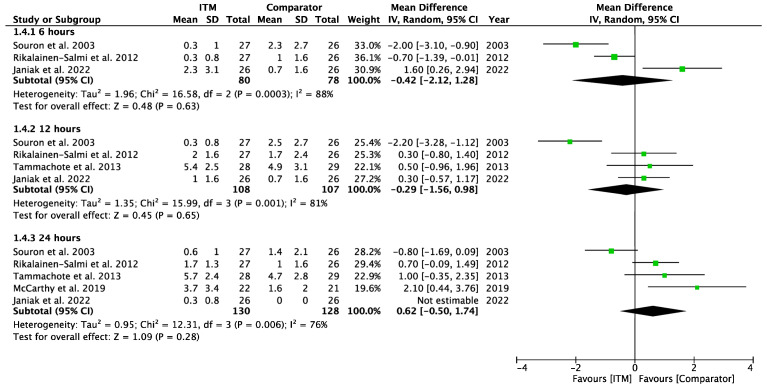
Postoperative Pain at Rest—Regional Comparators [15,26,28,29,30].

**Figure A6:**
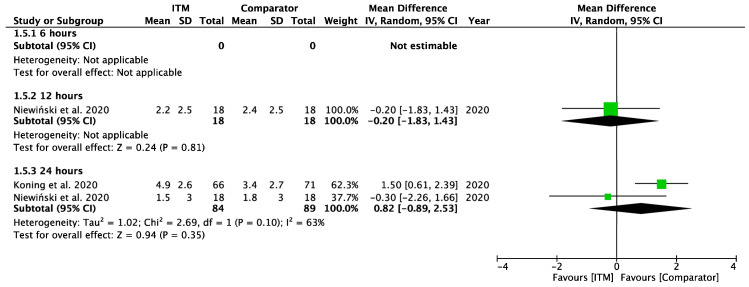
Postoperative Pain on Movement—Systemic Comparators [28].

**Figure A7:**
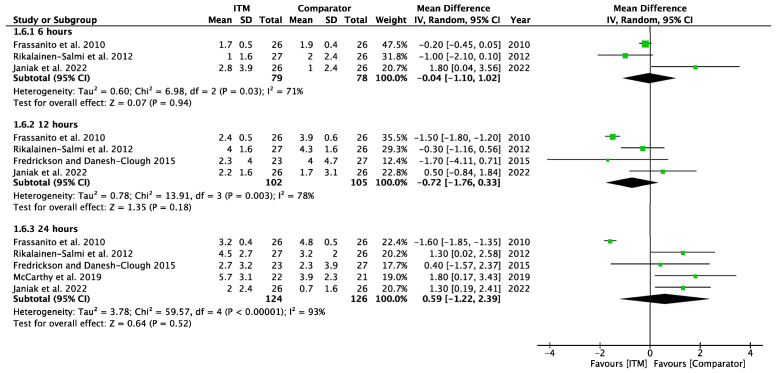
Postoperative Pain on Movement—Regional Comparators [14,15,24,27,30].

**Figure A8:**
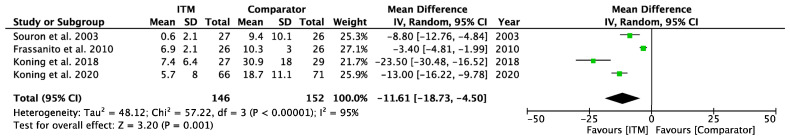
Cumulative Opioid Consumption [23,24,26,29].

**Figure A9:**
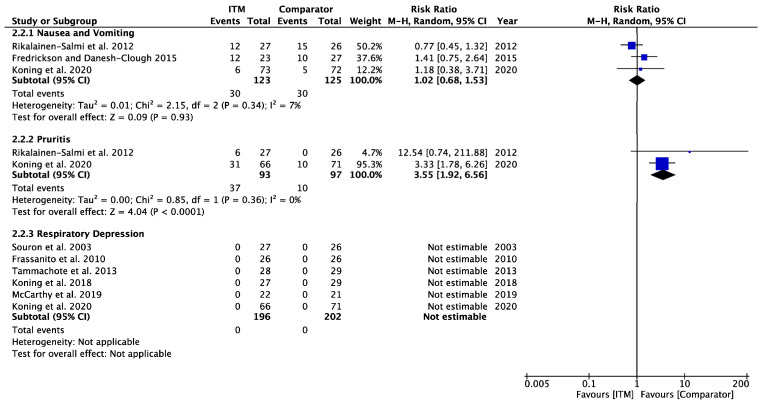
Opioid-Related Side Effects [14,15,23,24,27,28,29,30].

## Supplementary Materials

The following supporting information can be downloaded at: https://www.mdpi.com/article/10.3390/ph18040512/s1. Figure S1: Sensitivity Analysis of Postoperative Pain at Rest—includes only studies from most recent 10 years up to time of literature search; Figure S2: Sensitivity Analysis of Postoperative Pain on Movement—includes only studies from most recent 10 years up to time of literature search; Figure S3: Sensitivity Analysis of Postoperative Pain at Rest—Regional Comparators—includes only studies from most recent 10 years up to time of literature search; Figure S4: Sensitivity Analysis of Postoperative Pain on Movement—Regional Comparators—includes only studies from most recent 10 years up to time of literature search; Figure S5: Sensitivity Analysis of Cumulative Opioid Consumption—includes only studies from most recent 10 years up to time of literature search; Figure S6: Sensitivity Analysis of Opioid-Related Side Effects—includes only studies from most recent 10 years up to time of literature search.

## Appendix A

### Appendix A.1

Population: Adults (18 years and over) undergoing a surgical procedure

Intervention: Intrathecal morphine ± spinal anaesthesia

Control: Active comparator administered for the management of postoperative pain

Outcomes: Postoperative pain scores over first 24 h

Study: Randomised controlled trials

Databases searched: OVID Medline, Embase, APA PsycInfo, PubMed Central.

Search terms: ((intrathecal* adj3 morphine*) OR (spinal* adj3 morphine*) AND surger*) NOT (c*esarean* OR obstetric*)

  “adj3” only includes search results where these words appear within 3 word of one another

  “*” includes all words containing these characters

Limits: randomised controlled trials, adulthood (18+ years), human, English, last 20 years.

### Appendix A.2

Forest plot demonstrating meta-analysis of postoperative pain scores at rest within 24 h, with subgroup analysis at 6 h, 12 h, and 24 h and subgroup analysis of systemic and regional techniques compared to intrathecal morphine at 24 h. Bold text indicates title and subtotal rows.

### Appendix A.3

Forest plot demonstrating meta-analysis of postoperative pain sores on movement within 24 h, with subgroup analysis at 6 h, 12 h, and 24 h and subgroup analysis of systemic and regional techniques compared to intrathecal morphine at 24 h. Bold text indicates title and subtotal rows.

### Appendix A.4

Forest plot demonstrating sub-group meta-analysis of postoperative pain scores at rest at 6, 12, and 24 h in trials comparing systemic analgesia to intrathecal morphine. Bold text indicates title and subtotal rows.

### Appendix A.5

Forest plot demonstrating sub-group meta-analysis of postoperative pain scores at rest at 6, 12, and 24 h in trials comparing regional analgesia to intrathecal morphine. Bold text indicates title and subtotal rows.

### Appendix A.6

Forest plot demonstrating sub-group meta-analysis of postoperative pain scores on movement at 6, 12, and 24 h in trials comparing systemic analgesia to intrathecal morphine. Bold text indicates title and subtotal rows.

### Appendix A.7

Forest plot demonstrating sub-group meta-analysis of postoperative pain scores on movement at 6, 12, and 24 h in trials comparing regional analgesia to intrathecal morphine. Bold text indicates title and subtotal rows.

### Appendix A.8

Forest plot demonstrating meta-analysis of secondary outcome, cumulative opioid consumption, between ITM and active comparator groups in the first 24 h postoperatively. Bold text indicates title rows.

### Appendix A.9

Forest plot demonstrating meta-analysis of secondary outcome and opioid-related side effects, between ITM and active comparator groups in the first 24 h postoperatively. Bold text indicates title and subtotal rows.
