# Supplementary figures and images for: Intrathecal Morphine Versus Other Techniques for Postoperative Pain Management in the Context of Multimodal Analgesia: A Meta-Analysis
Source: Pharmaceuticals (Basel). 2025 Mar 31;18(4):512. doi: 10.3390/ph18040512 (PMC12030641; doi:10.3390/ph18040512)

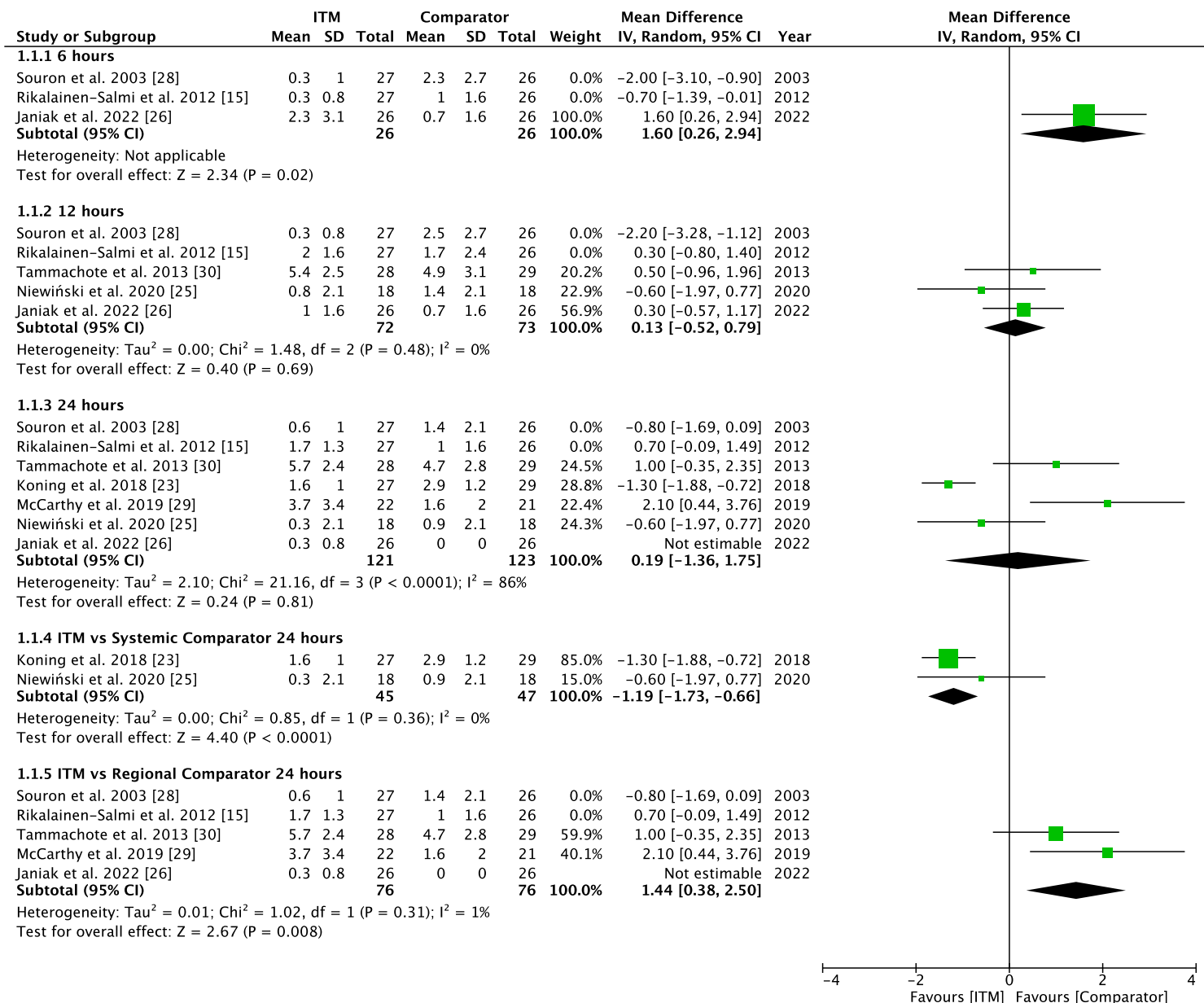

Supplement: Supplementary file 1 [file pharmaceuticals-18-00512-s001.zip › Sensitivity Analysis/Figure S1.pdf]

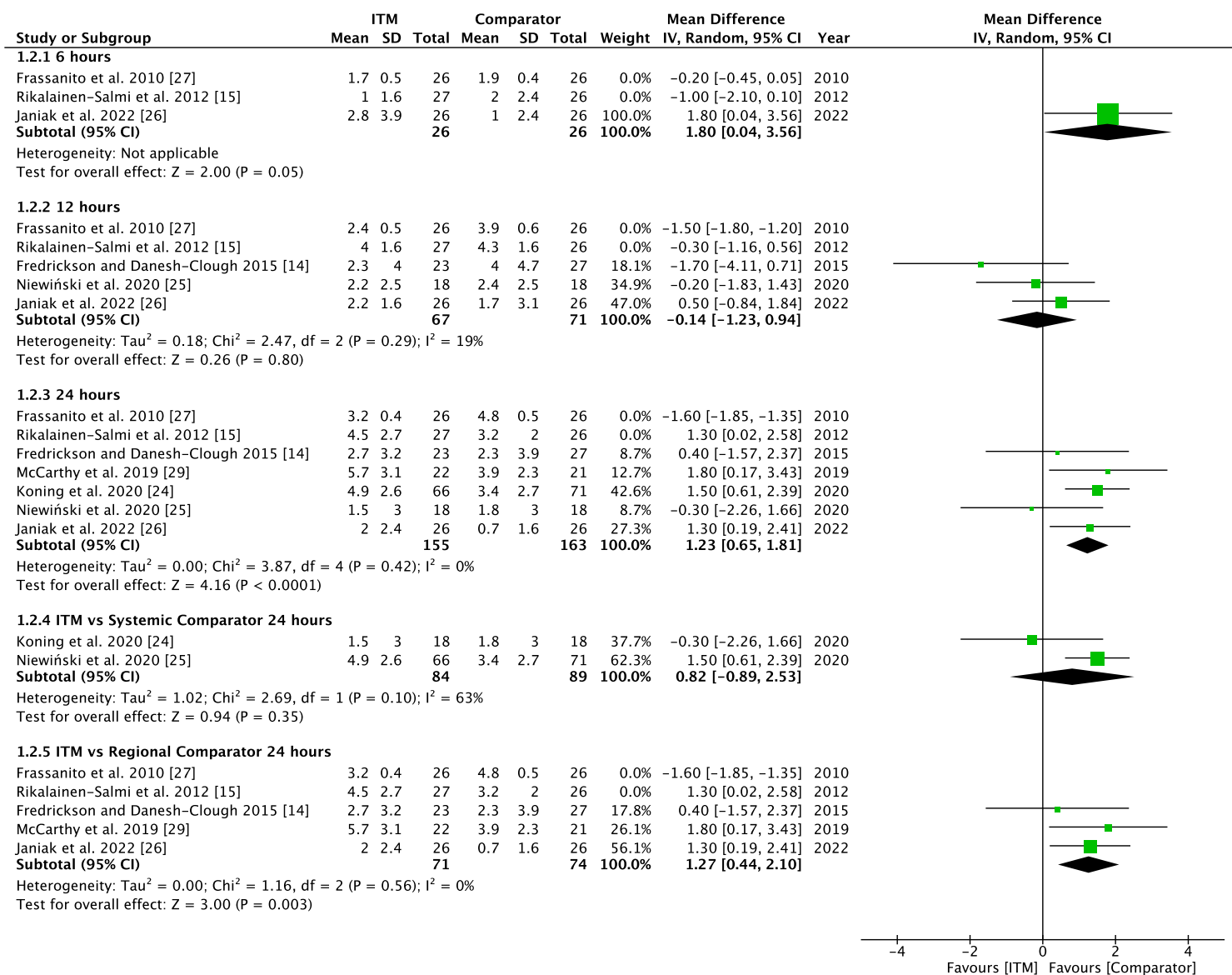

Supplement: Supplementary file 1 [file pharmaceuticals-18-00512-s001.zip › Sensitivity Analysis/Figure S2.pdf]

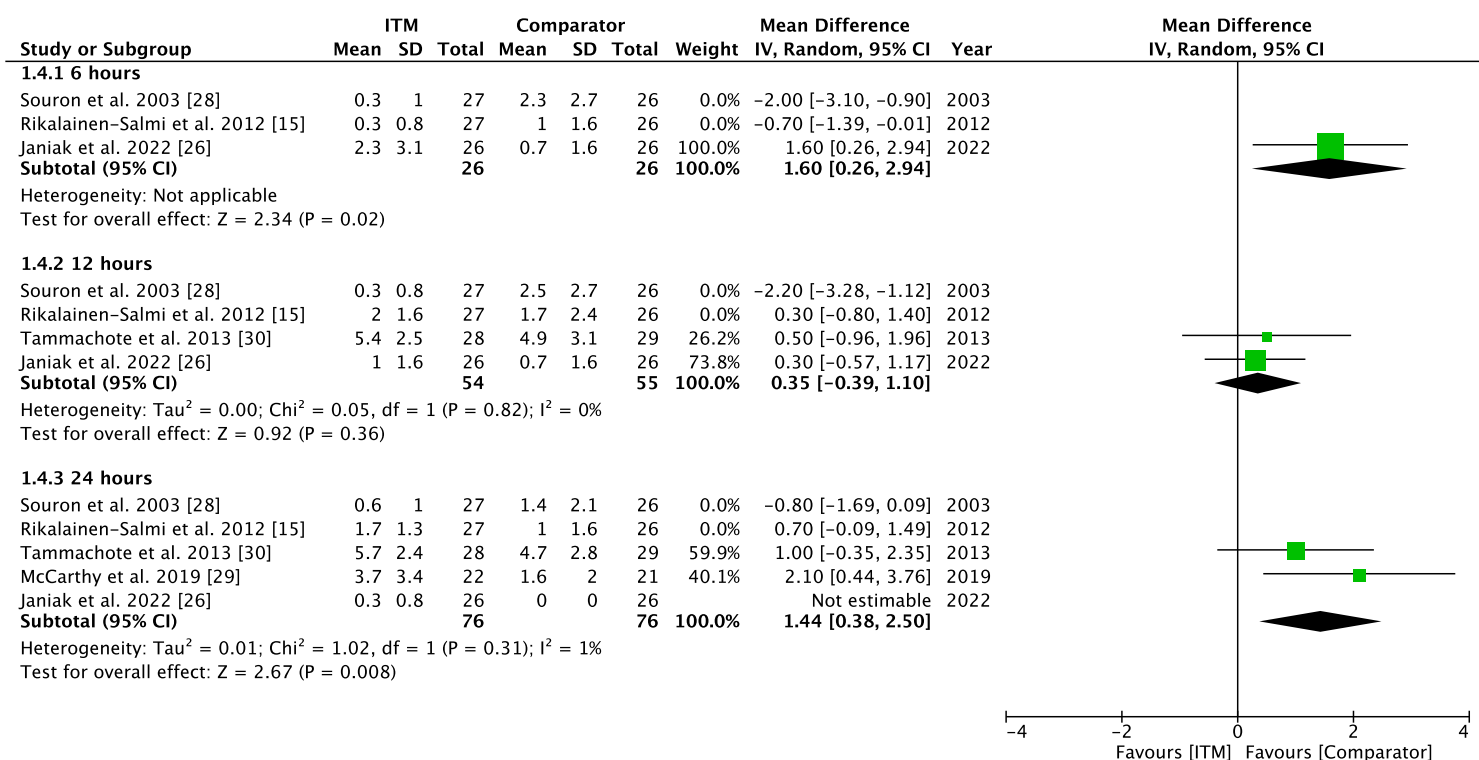

Supplement: Supplementary file 1 [file pharmaceuticals-18-00512-s001.zip › Sensitivity Analysis/Figure S3.pdf]

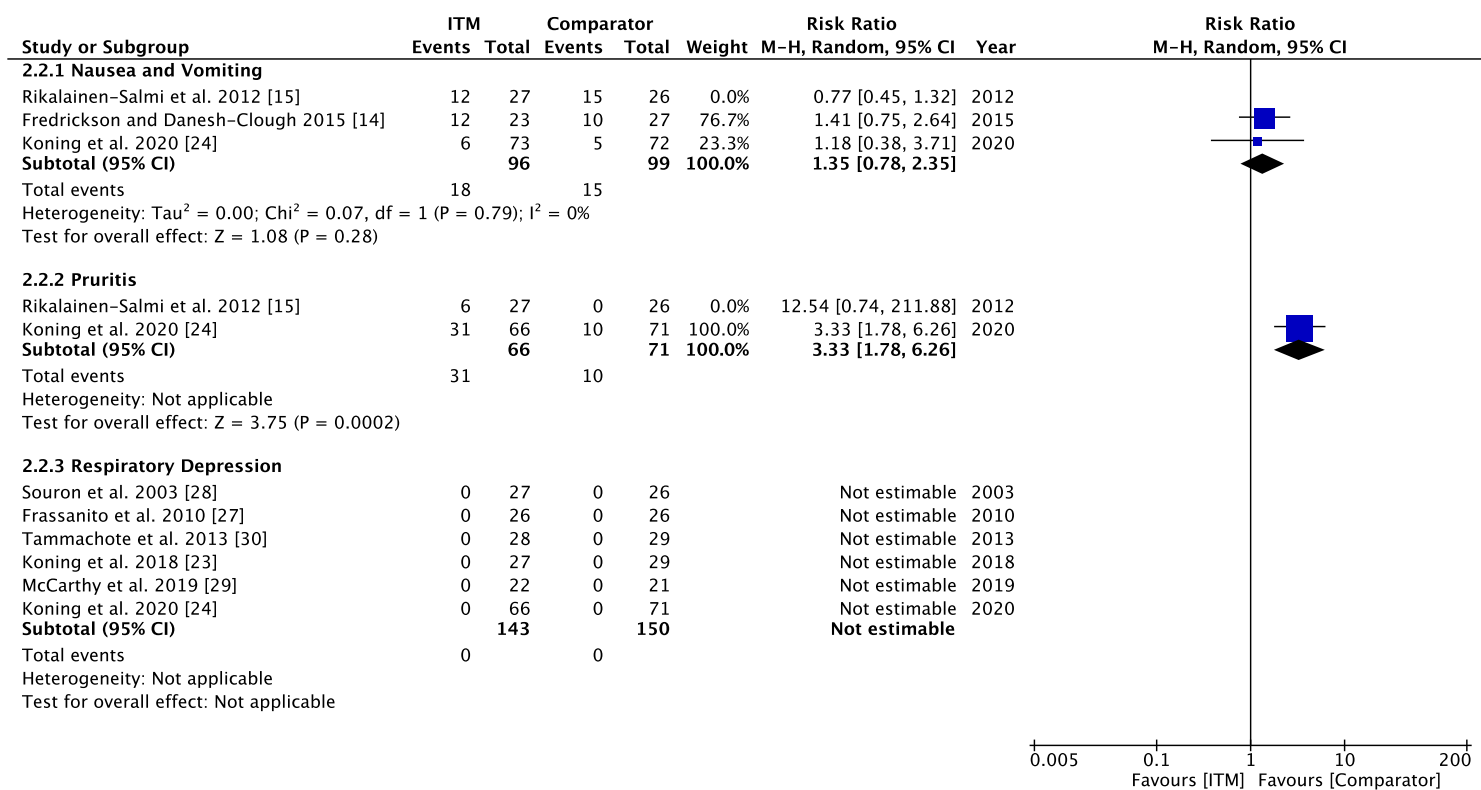

Supplement: Supplementary file 1 [file pharmaceuticals-18-00512-s001.zip › Sensitivity Analysis/Figure S6.pdf]

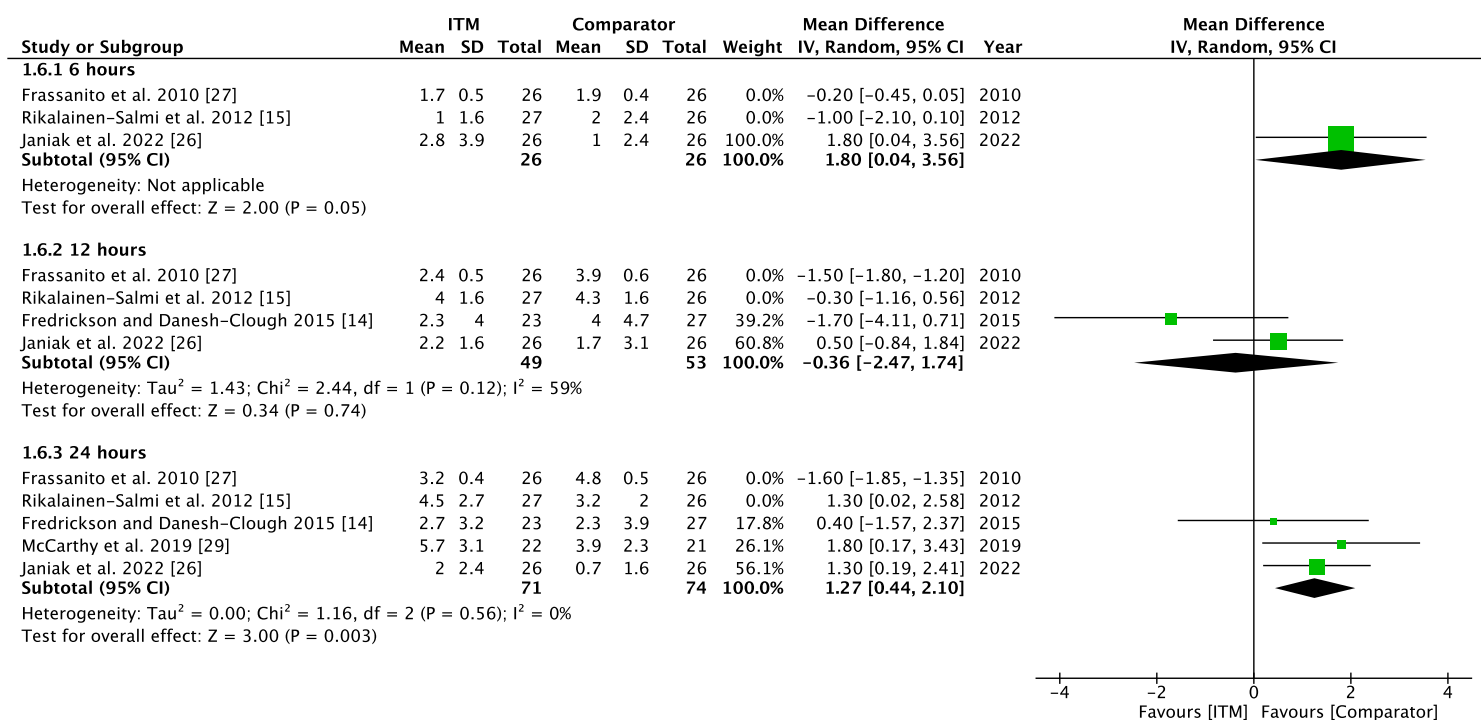

Supplement: Supplementary file 1 [file pharmaceuticals-18-00512-s001.zip › Sensitivity Analysis/Figure S4.pdf]

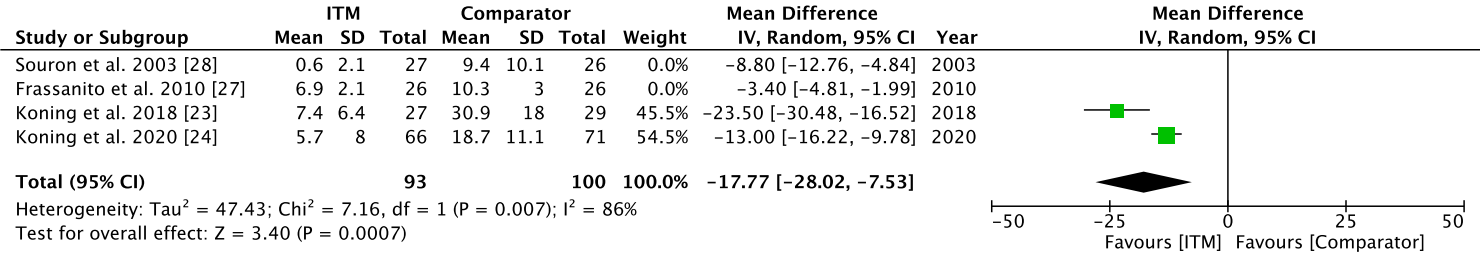

Supplement: Supplementary file 1 [file pharmaceuticals-18-00512-s001.zip › Sensitivity Analysis/Figure S5.pdf]
